# Acute caffeine strategies for improving performance in combat sports: a structured narrative review

**DOI:** 10.3389/fphys.2026.1877799

**Published:** 2026-09-10

**Authors:** Jingang Fan, Yike Li, Dongchen Li, Hansen Li, Guodong Zhang

**Affiliations:** 1 Department of Physical Education, Sichuan International Studies University, Chongqing, China; 2 College of Physical Education, Southwest University, Chongqing, China; 3 School of Physical Education and Health, Yili Normal University, Yili, Xinjiang, China; 4 School of Physical Education, Sichuan Agricultural University, Ya’an, China; 5 International College, Krirk University, Bangkok, Thailand

**Keywords:** acute caffeine supplementation, caffeine, combat sports, combined intervention, ergogenic aid, exercise performance

## Abstract

**Objective:**

This structured narrative review updates and contextualizes evidence on acute caffeine strategies in combat sports, with emphasis on performance effects, delivery form, combined interventions, and factors that may explain inconsistent findings.

**Methods:**

A structured literature search was conducted in Web of Science Core Collection, Scopus, PubMed, and SPORTDiscus. Eligible reports involved trained amateur, competitive, or professional combat sport athletes, evaluated an acute caffeine intervention administered before or during exercise or sport specific performance testing, and included at least one physical, cognitive, or technical tactical performance outcome. Findings were synthesized narratively because of substantial heterogeneity in sport discipline, caffeine protocol, comparator, and outcome measures.

**Results:**

Forty three reports met the eligibility criteria. Fourteen evaluated combined caffeine protocols, whereas 29 evaluated caffeine supplementation alone. Combined protocols included caffeine with sodium bicarbonate, carbohydrate, L theanine, Rhodiola rosea, warm up music, conditioning activity, post activation performance enhancement, or mouth rinsing. These strategies showed potential context specific benefits, particularly when caffeine was combined with warm up music or conditioning activity, but they did not establish a general advantage over isolated caffeine supplementation. For caffeine supplementation alone, favorable findings were reported for selected simulated combat outcomes, Special Judo Fitness Test performance, strength, power, muscular endurance, reaction related outcomes, and repeated high intensity activity. However, null findings were also common, even within commonly used doses of 3 to 6 mg/kg. Perceived exertion and heart rate were generally unchanged, whereas blood lactate more frequently increased following caffeine ingestion.

**Conclusions:**

Acute caffeine supplementation may improve selected performance outcomes in combat sport athletes, but responses vary across disciplines, tasks, athlete characteristics, dosing strategies, delivery forms, and competition formats. Doses of 3 to 6 mg/kg remain the most frequently studied protocols, but individual responses should be assessed during training before competition. Evidence for high or repeated doses, rapid delivery forms, and combined strategies remains preliminary.

## 1 Introduction

Combat sports include various contact disciplines such as boxing, judo, Brazilian Jiu-Jitsu (BJJ), karate, taekwondo, and mixed martial arts (MMA) (Barley et al., 2019). Although their rules and bout structures differ, these sports share repeated high-intensity actions separated by lower-intensity movement and brief recovery. Performance therefore depends on maximal strength, explosive power, muscular endurance and the capacity to repeat glycolytic and phosphagen-dominant efforts (Campos et al., 2012; da Silva et al., 2015; Franchini et al., 2019; Vasconcelos et al., 2020). Moreover, rapid recovery is also a key factor in athletic performance (Ghoul et al., 2019). Rapid weight-loss practices, close-contact decision making and tournament formats involving several bouts on the same day further distinguish combat sports from other exercise. In tournaments with successive matches, this capacity determines whether athletes can maintain high-level performance and avoid fatigue-induced decline. At the elite level, where competition is extremely fierce, even minimal improvements may make a decisive difference in outcomes (Christensen et al., 2017). These demands make ergogenic aids potentially useful (Garthe and Maughan, 2018). Among these, caffeine stands out as one of the most commonly consumed ergogenic aids among athletes. Reports indicate that more than three-quarters of athletes consume caffeine prior to or during competition (Pickering and Kiely, 2019), and the amount ingested by athletes frequently exceeds that of their non-athletic peers, with habitual intake ranging from 300 to 700 mg per day (Bell and McLellan, 2002; Soeren and Graham, 1998).

Caffeine is naturally present in many food sources such as coffee, cola, tea, chocolate, and energy drinks (Peeling et al., 2018). After ingestion, caffeine levels in the blood begin to rise within 15–45 minutes and peak around 60 minutes (López-González et al., 2018). Caffeine has been reported to improve endurance performance and resistance to fatigue (Graham, 2001), reduce early-phase glycogen breakdown, and prolong exercise duration (Spriet et al., 1992). Evidence also suggests that acute caffeine supplementation may improve upper-body strength (Beck et al., 2006; Woolf et al., 2008). A meta-analysis found favorable effects of caffeine on aerobic endurance, muscular strength and endurance, explosive power, jumping performance, and movement speed (Grgic et al., 2020). In closed high-intensity endurance efforts lasting 45 seconds to 8 minutes, caffeine has been reported to improve average speed (Christensen et al., 2017). Benefits of caffeine have also been observed in improving reaction time (Główka et al., 2024; Santos et al., 2014; Souissi et al., 2013), potentially due to its effects on the neurotransmitter dopamine—prolonging its activity and increasing arousal levels (Torres and Kim, 2019). Caffeine may also enhance alertness and vigilance, boost cognitive function, and improve attention and memory (Glade, 2010; Hogervorst et al., 2008, 1999; Kennedy and Scholey, 2004). These caffeine-induced improvements are considered critical determinants of performance outcomes in combat sports. According to Aguilar-Navarro et al. (2019), the proportion of Olympic athlete urine samples testing positive for caffeine increased from 70% in 2004 to 75% in 2015, with notable increases in caffeine concentrations found particularly in boxing and judo. It is important to note that the ergogenic effects of caffeine exhibit substantial inter-individual variability, influenced by a wide range of factors such as habitual intake, genotype, sex, training status, time of day, expectancy, and delivery form (Pickering and Kiely, 2018).

Previous reviews and meta analyses have shown that caffeine may improve some performance outcomes in combat sports (Delleli et al., 2022; Diaz-Lara et al., 2023; López-González et al., 2018). Since then, the evidence base has expanded, with newer studies testing different delivery forms, combined strategies, repeated dose protocols, and more sport specific performance tasks. However, the findings remain mixed, suggesting that caffeine effects may depend on the sport, testing task, athlete characteristics, and competition format. Therefore, the present structured narrative review updates the evidence through July 2026, separates isolated caffeine supplementation from combined or mixed intervention protocols, and discusses factors that may contribute to the inconsistent findings. This practical synthesis complements previous meta analyses by linking study results to the specific demands of combat sport competition.

## 2 Methods

We conducted a structured narrative review using transparent procedures for the literature search, study selection, data extraction, and narrative synthesis, consistent with recommendations for improving the rigor and transparency of narrative reviews (Ferrari, 2015; Paré et al., 2015).

### 2.1 Information sources and search strategy

The original literature search was conducted in the Web of Science Core Collection and Scopus from database inception to 24 January 2025. During revision, the searches in these databases were updated, and database coverage was expanded to include PubMed and SPORTDiscus. The final search was conducted on 31 July 2026.

The search strategy was informed by previous reviews (Li et al., 2025; Luo et al., 2025; Vasconcelos et al., 2020). The search combined the term “caffeine” with terms describing combat sports and specific disciplines: caffeine AND (combat sports OR martial arts OR boxing OR wrestling OR judo OR taekwondo OR karate OR kickboxing OR muay thai OR Brazilian jiu-jitsu OR MMA OR “mixed martial arts”). The search syntax was adapted to the requirements of each database.

### 2.2 Eligibility criteria

Eligible reports described primary studies involving trained amateur, competitive, or professional athletes participating in a recognized combat sport. No age restriction was applied. Studies were required to evaluate an acute caffeine intervention administered before or during exercise or sport-specific performance testing. Caffeine could be administered alone or as part of a combined or mixed-intervention protocol.

Eligible comparators included placebo, a non-caffeine control, another caffeine dose or delivery form, or the same co-intervention without caffeine. At least one physical, cognitive, or technical-tactical performance outcome was required. Physiological and perceptual outcomes were considered only when reported alongside an eligible performance outcome. Reviews, non-English publications, and reports without separately extractable data for an eligible subgroup of combat sports athletes were excluded.

### 2.3 Study selection and data extraction

**Figure 1:**
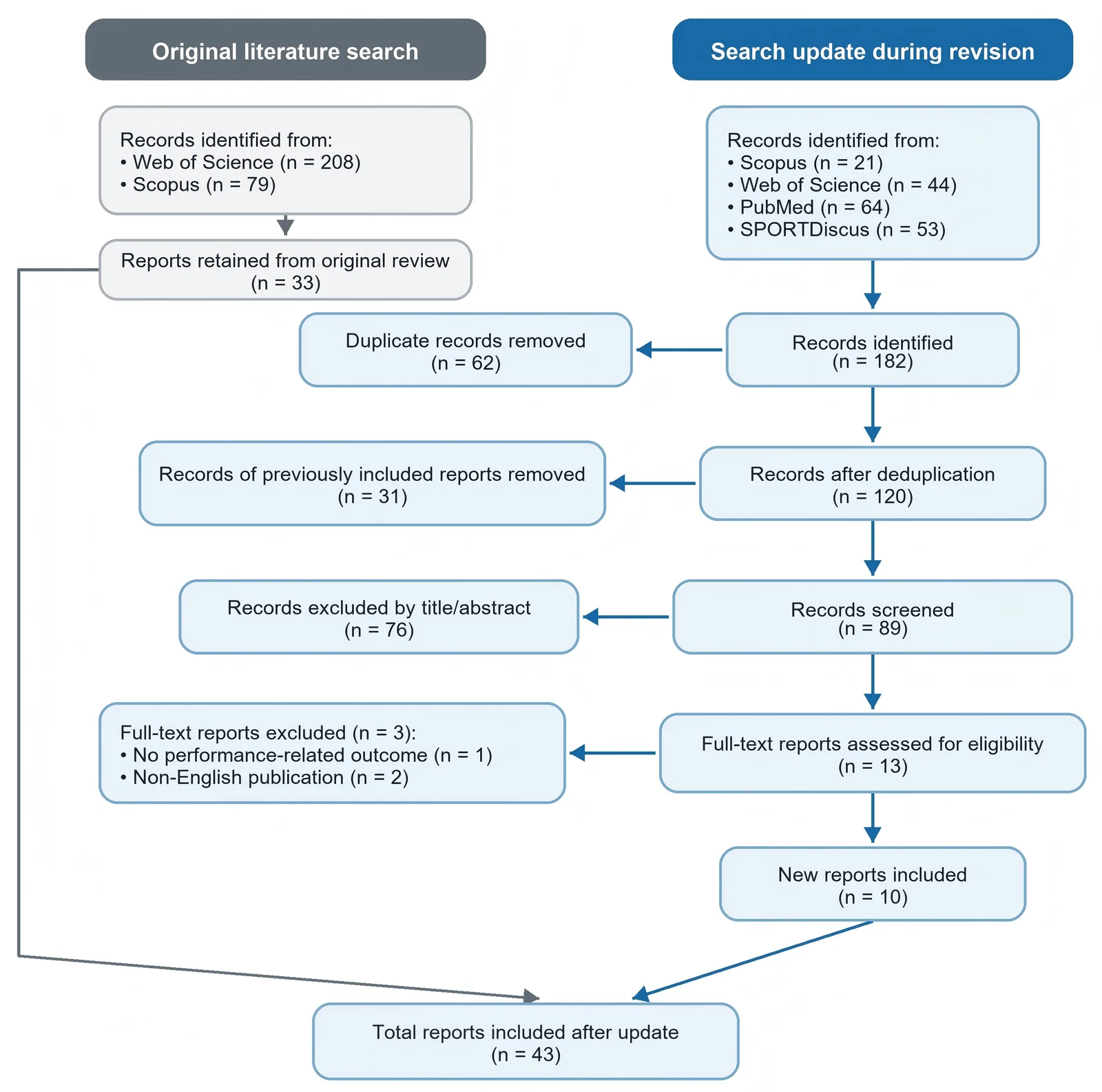
Flow of report identification and inclusion during the original and updated searches.

Two authors (Jingang Fan and Yike Li) independently screened titles and abstracts before assessing the full texts of potentially eligible reports. Disagreements were resolved by consensus with a third author (Hansen Li). For each included report, data were extracted on the author and year, participant characteristics, study design, caffeine dose, delivery form, timing, comparator, outcomes assessed, and key findings. **Figure 1** presents the study-selection process, record counts, and grouped reasons for full-text exclusion.

### 2.4 Narrative synthesis

Because the included studies differed substantially in design, caffeine protocol, comparator, and performance-related outcomes, their findings were synthesized narratively. Combined or mixed-intervention studies were synthesized separately from caffeine-only studies. Within the caffeine-only group, the tabulated evidence was organized by combat-sport discipline, whereas the narrative synthesis was organized by outcome domain. Findings were subsequently compared across caffeine dose, delivery form, timing, comparator, and outcome domain to identify consistent patterns, conflicting results, and potential sources of heterogeneity. Because the aim was to provide a contextual narrative synthesis rather than quantitative effect estimates or formal grading of evidence certainty, no formal risk-of-bias or certainty-of-evidence instrument was applied.

## 3 Results

The original search, completed on 24 January 2025, identified 287 records, including 208 from the Web of Science Core Collection and 79 from Scopus. Following deduplication, screening, and full-text eligibility assessment, 33 reports were retained. The updated and expanded search, completed on 31 July 2026, identified 182 records, comprising 64 from PubMed, 44 from the Web of Science Core Collection, 21 from Scopus, and 53 from SPORTDiscus. Ten additional reports met the eligibility criteria, resulting in a total of 43 reports in the updated review (**Figure 1**).

Of the 43 included reports, 14 evaluated combined caffeine protocols (**Table 1**), whereas 29 evaluated caffeine supplementation alone (**Table 2**). To facilitate comparison across heterogeneous findings, the evidence was first organized by intervention type. Caffeine-only evidence was tabulated by combat-sport discipline and synthesized narratively by outcome domain.

**Table 1:** Characteristics and key findings of reports evaluating combined caffeine protocols.

Study/report	Participants and design	Intervention/comparator	Outcomes assessed	Key findings
Delleli et al. (2023)	16 elite male taekwondo athletes; double-blind, randomized, placebo-controlled crossover	CAF 3 mg/kg, placebo, or no supplement, each with or without preferred warm-up music (six conditions)	TSAT; FSKT-10s; FSKT-mult; RPE; affect and enjoyment	CAF plus music improved all three taekwondo-specific tests versus the other conditions and produced generally more favorable perceptual and affective responses.
Delleli et al. (2024a)	16 elite female taekwondo athletes; double-blind, randomized, placebo-controlled crossover	CAF 3 mg/kg, placebo, or no supplement, each with or without preferred warm-up music (six conditions)	Simulated-combat activity; technical–tactical actions; HR; RPE; affect and enjoyment	CAF plus music increased attack time and offensive, counter-attacking, and defensive actions, reduced HRmean and HRpeak, and produced more favorable perceptual and affective responses than most other conditions.
Delleli et al. (2024b)	16 elite female taekwondo athletes; double-blind, randomized, placebo-controlled crossover	CAF 3 mg/kg, placebo, or no supplement, each with or without preferred warm-up music (six conditions)	TSAT; FSKT-10s; FSKT-mult; RPE; affect and enjoyment	CAF plus music improved FSKT-10s and FSKT-mult performance and several perceptual and affective outcomes. CAF plus music and CAF alone both improved TSAT.
Delleli et al. (2025)	16 elite male taekwondo athletes; double-blind, randomized, counterbalanced crossover	CAF 3 mg/kg, placebo, or no supplement, each with or without preferred warm-up music (six conditions)	Simulated-combat activity; technical–tactical actions; HR; RPE; affect and enjoyment	CAF plus music extended attack time, increased offensive and defensive actions, and improved several affective responses compared with the single-strategy conditions. It also reduced HRmean and HRpeak versus the other conditions.
Ouergui et al. (2022)	20 young taekwondo athletes (10 male, 10 female); double-blind, randomized, counterbalanced crossover	CAF 3 mg/kg or placebo, with or without a conditioning activity; no-intervention control	TSAT; FSKT-10s; FSKT-mult; RPE and psychological measures	CAF plus the conditioning activity improved TSAT and both FSKT outcomes versus all other conditions.
Felippe et al. (2016)	10 experienced male judokas; double-blind, randomized, counterbalanced crossover	CAF 6 mg/kg and/or NaHCO_3_ 0.3 g/kg versus placebo	Three repeated SJFTs; total throws; blood lactate; RPE	Only CAF plus NaHCO_3_ increased total throws across the three SJFTs versus placebo. Superiority over either supplement alone was not established.
Rezaei et al. (2019)	8 competitive karate athletes; double-blind, randomized, placebo-controlled crossover	CAF 6 mg/kg and/or NaHCO_3_ 0.3 g/kg versus placebo and control	Karate-specific aerobic test time to exhaustion; blood lactate; RPE	CAF, NaHCO_3_, and their combination prolonged time to exhaustion versus placebo and control, with no difference among the three active conditions.
Zhang et al. (2024)	25 highly trained male boxers; double-blind, randomized crossover	PAPE plus CAF 3 mg/kg, PAPE plus placebo, or control without PAPE/supplement	30-s Wingate peak and mean power; total work; post-test blood lactate; HR; RPE; perceived power	Both PAPE conditions improved Wingate mean power and total work versus control. PAPE plus CAF also increased peak power, post-test blood lactate, and perceived power versus control; however, no variables differed directly between PAPE plus CAF and PAPE plus placebo.
Wang et al. (2026)	15 male amateur boxers; double-blind, randomized, placebo-controlled crossover	Caffeinated gum (0, 3, or 6 mg/kg) with or without bench-press PAPE	Bench-press mean and peak bar velocity; total repetitions	PAPE and caffeinated gum independently improved bench-press bar velocity. PAPE plus caffeinated gum enhanced bar velocity compared with caffeinated gum alone; however, caffeine increased total repetitions only when used without PAPE.
Suksuwan et al. (2022)	28 combat sports (boxing, kabaddi, and karate) athletes; randomized, single-blind, crossover	CAF 175 mg plus 12% CHO versus 12% CHO	Isokinetic knee strength; vertical jump; Wingate anaerobic performance	CAF plus CHO increased knee-extension peak torque and relative Wingate mean power; vertical-jump height, relative peak power, and fatigue index did not differ.
Razazan et al. (2025)	12 elite male wrestlers; double-blind, randomized, placebo-controlled crossover	CAF 3 mg/kg, L-theanine 3 mg/kg, CAF plus L-theanine 3 mg/kg each, or placebo	Wall-squat test; VJH; MBT; handgrip strength; SWFT; Stroop performance; STAI anxiety; side effects	CAF plus L-theanine improved wall-squat time, MBT, VJH, and grip strength versus placebo and produced the highest SWFT throw count across conditions. It also improved post-SWFT Stroop reaction time and accuracy, while reducing anxiety and caffeine-related tachycardia compared with CAF alone.
Tao et al. (2025)	48 trained boxers (12 per group); double-blind, randomized, placebo-controlled parallel trial	8 weeks of Rhodiola rosea 2.4 g/day, acute CAF 3 mg/kg 30 min before final testing, both supplements, or control	Straight-punch force and velocity; ground-reaction force; 30s continuous punching test	CAF plus Rhodiola rosea improved straight-punch force/velocity, ground-reaction-force variables, and 30-s punching count/power versus control and selected single-supplement conditions.
Özlükan-Şahin et al. (2024)	16 trained male karate athletes; double-blind, placebo-controlled crossover	6.4% CHO, 1.2% CAF, or water mouth rinse	Repeated-sprint power; kick force, power, duration, and reaction time; hand reaction time; HR; RPE	CAF showed the highest numerical relative sprint power and fastest second-set reaction times, but these outcomes did not differ significantly from placebo water; significant differences were mainly versus CHO.
Pak et al. (2020)	27 competitive taekwondo athletes; counterbalanced crossover during Ramadan	CAF mouth rinse (6 mg/kg), 6.4% glucose mouth rinse, or placebo mouth rinse	Taekwondo Anaerobic Intermittent Kick Test; successful kicks; RPE	The CAF rinse increased the percentage of successful kicks versus glucose and placebo during the first three weeks of Ramadan; RPE was not significantly reduced.

For Tao et al. (2025), only data from the subgroup of trained boxers (n = 48) were extracted because the untrained subgroup (n = 48) did not meet the eligibility criteria.

CAF, caffeine; CHO, carbohydrate; FSKT-10s, 10-s Frequency Speed of Kick Test; FSKT-mult, multiple Frequency Speed of Kick Test; HR, heart rate; MBT, medicine-ball throw; NaHCO_3_, sodium bicarbonate; PAPE, post-activation performance enhancement; RPE, rating of perceived exertion; SJFT, Special Judo Fitness Test; STAI, State-Trait Anxiety Inventory; SWFT, Specific Wrestling Fitness Test; TSAT, Taekwondo-Specific Agility Test; VJH, vertical jump height.

**Table 2:** Characteristics and key findings of reports evaluating caffeine supplementation alone.

Study/report	Participants and design	Intervention/comparator	Outcomes assessed	Key findings
Judo				
Souissi et al. (2012)	12 elite judokas; randomized, double-blind crossover	CAF 5 mg/kg versus placebo, 60 min before morning testing at approximately 07:00	Simple reaction time; mood states; 30-s Wingate peak and mean power and fatigue index	CAF reduced simple reaction time and increased Wingate peak and mean power; the fatigue index was unchanged. Anxiety and vigor increased.
Lopes-Silva et al. (2014)	6 experienced male judokas; double-blind, counterbalanced crossover, with each trial conducted after a 5-day rapid-weight-loss period	CAF 6 mg/kg versus placebo, 60 min before testing	Three repeated SJFTs (throws and index); RPE; HR; plasma lactate	CAF did not improve SJFT throws or index after rapid weight loss, but reduced RPE and increased plasma lactate; HR was unchanged.
Souissi et al. (2015)	10 elite judokas; randomized, double-blind crossover	CAF 5 mg/kg versus placebo, 60 min before afternoon testing at approximately 17:00	Simple reaction time; mood states; 30-s Wingate peak and mean power and fatigue index	CAF reduced simple reaction time but did not affect Wingate peak power, mean power, or fatigue index. Anxiety and vigor increased.
Astley et al. (2017)	18 male judokas; randomized, double-blind crossover	CAF 4 mg/kg versus placebo, 60 min before testing	SJFT throws and index; RPE; HR	CAF increased throws and reduced the SJFT index and RPE compared with placebo; HR was unchanged.
Saldanha Da Silva Athayde et al. (2018)	14 male judokas; randomized, double-blind crossover	CAF 5 mg/kg versus placebo, 60 min before testing	CMJ; handgrip strength; JGST; simulated matches; blood lactate	CAF increased blood lactate but did not improve CMJ performance, handgrip strength, JGST performance, or the number of attacks.
Durkalec-Michalski et al. (2019)	22 highly trained male judokas; randomized, double-blind, placebo-controlled crossover	CAF 3, 6, or 9 mg/kg versus placebo, 60 min before testing	SJFT total throws and index; attacks during three randori bouts; HR; RPE	CAF 6 and 9 mg/kg increased total SJFT throws and post-SJFT HR; 9 mg/kg also increased combat activity. SJFT index and RPE were unchanged.
Saldanha Da Silva Athayde et al. (2019)	12 male judokas; randomized, double-blind crossover	CAF 5 mg/kg versus placebo, 60 min before testing	Attacks, efficiency, and effectiveness during simulated matches; RPE; RPR	CAF did not affect attacks, efficiency, effectiveness, RPE, or RPR.
Carmo et al. (2021)	8 high-level judokas; controlled, randomized, crossover, double-blind	CAF 5 mg/kg versus placebo, 60 min before testing	Pre- and post-training SJFT performance, CMJ, and upper-limb power; HR and RPE	CAF maintained SJFT throws and index after training, whereas placebo was associated with fewer throws and a higher index. CAF did not affect CMJ, upper limb power, HR, or RPE.
Filip-Stachnik et al. (2021)	9 elite male Polish national-team judokas; randomized, double-blind, placebo-controlled crossover	Caffeinated gum (200 or 400 mg total) versus non-caffeinated gum; one piece consumed 15 min before each of two SJFTs	Two repeated SJFTs (throws and index); RPE; HR; blood lactate	Neither caffeine dose improved throws, SJFT index, RPE, HR, or blood lactate compared with placebo.
Krawczyk et al. (2022)	10 highly trained national-level judokas (6 men, 4 women); randomized, double-blind, placebo-controlled crossover	CAF 3 or 6 mg/kg versus placebo, 60 min before testing	Bench-press and bench-pull; CMJ; handgrip strength; dynamic and isometric JGST	Both doses increased bench press peak velocity, bench pull mean velocity, and dynamic JGST repetitions. Only 6 mg/kg increased bench press mean velocity; neither dose improved jump height, maximal handgrip strength, or isometric JGST duration.
Taekwondo				
Santos et al. (2014)	10 male taekwondo athletes; randomized, double-blind, repeated-measures crossover	CAF 5 mg/kg versus placebo; reaction-time testing began 50 min after ingestion, followed by two simulated combats separated by 20 min (first combat at 60 min)	Reaction time, simulated taekwondo contest; HR; RPE; plasma lactate	CAF reduced reaction time before the first combat and helped maintain combat activity across successive combats; plasma lactate was higher during rounds 2 and 3 of the first combat. Kick time, HR, RPE, and scores were unchanged.
Lopes-Silva et al. (2015)	10 high-level male taekwondo athletes; randomized, double-blind, repeated-measures crossover	CAF 5 mg/kg versus placebo, 60 min before testing	Simulated combat; estimated energy-system contributions; HR; RPE; postexercise parasympathetic reactivation	CAF increased peak blood lactate and estimated glycolytic contribution but did not affect combat performance, HR, RPE, or postexercise parasympathetic reactivation.
Sun et al. (2022)	10 elite taekwondo athletes (6 men, 4 women); randomized, double-blind crossover	CAF 200 mg versus placebo drink, 60 min before testing	Simulated match; WAnT; EFT, Stroop, and RVIPT performance; HR; RPE; blood lactate	CAF increased WAnT peak power and relative peak and mean power, and shortened pre-exercise EFT reaction time. Simulated-match and cognitive-test accuracy were unchanged.
Ouergui et al. (2023)	52 young taekwondo athletes (26 men, 26 women; elite and sub-elite); randomized, double-blind, placebo-controlled crossover	CAF 3 mg/kg versus placebo and no-treatment control, 60 min before testing	TSAT; FSKT-10s; FSKT-multi; s-RPE	CAF improved TSAT and FSKT-10s in elite and sub-elite athletes and improved FSKT-multi mainly in elite athletes; s-RPE decreased only in elite athletes.
Nana et al. (2025)	13 male university-level taekwondo athletes; randomized, double-blind crossover	CAF 200 mg versus placebo, 60 min before testing	EEG activity; auditory P300 ERP; cognitive performance; TSAT; HR; RPE; blood glucose; blood lactate	CAF transiently reduced delta-wave activity before fatigue and increased P300 amplitude after fatigue; reaction time, accuracy, TSAT performance, and physiological responses were unchanged.
Jiu-jitsu				
Diaz-Lara et al. (2016a)	14 elite male BJJ athletes; randomized, double-blind, placebo-controlled crossover	CAF 3 mg/kg versus placebo, 60 min before testing	Handgrip strength; CMJ; maximal static lift; bench press 1RM, power load, and repetitions to failure	CAF increased bilateral handgrip strength, CMJ height, maximal static lift duration, bench press 1RM and maximal power, and repetitions and mean power during the test to failure.
Diaz-Lara et al. (2016b)	14 elite male BJJ athletes; randomized, double-blind, placebo-controlled crossover	CAF 3 mg/kg versus placebo, 60 min before testing	simulated BJJ combats; maximal handgrip, CMJ, static-lift, and bench press tests; blood lactate; RPE	CAF increased offensive action time in both combats, blood lactate, and RPE. All pre-combat physical tests improved, with static-lift and bench press benefits remaining after combat 1; no physical-test differences remained after combat 2.
Merino-Fernández et al. (2021)	16 elite Spanish national-team traditional Jiu-jitsu athletes (8 men, 8 women); randomized, double-blind, placebo-controlled crossover	CAF 3 mg/kg versus placebo, 60 min before testing	Bilateral and unilateral CMJ performance	CAF increased bilateral CMJ performance and selected unilateral CMJ outcomes.
Lopes-Silva et al. (2022)	10 experienced athletes (2 judo, 8 Jiu-jitsu); double-blind, placebo-controlled crossover	CAF 5 mg/kg versus placebo, 60 min before testing	judogi dynamic strength endurance test; maximal isometric handgrip strength; HR; RPE; blood lactate	CAF increased total repetitions by 7% and maximal isometric handgrip strength by 5%. HR, RPE, and blood lactate did not differ between conditions.
Merino-Fernández et al. (2022)	22 young traditional Jiu-jitsu athletes (11 men, 11 women); randomized, double-blind, placebo-controlled crossover	CAF 3 mg/kg versus placebo, 60 min before testing	SJFT throws and index; HR; perceived strength, endurance, and fatigue; simulated-combat	CAF increased total throws, HR, perceived strength, and perceived endurance, and reduced perceived fatigue. The SJFT index was higher and combat actions were unchanged.
Wrestling				
Aedma et al. (2013)	14 trained male wrestlers; double-blind, counterbalanced crossover	CAF 5 mg/kg versus placebo, 30 min before testing	UBISP peak and mean power; blood lactate; HR; RPF; RPE	CAF did not improve UBISP performance and was associated with a decline in peak power across the four tests. HR and blood lactate were higher, whereas RPF and RPE were unchanged.
Negaresh et al. (2018)	12 professional male freestyle wrestlers; randomized, double-blind, counterbalanced and crossover	Placebo; CAF 4 or 10 mg/kg 45 min before match 1; repeated CAF 2 mg/kg before each match (10 mg/kg total); or individualized selective CAF dosing based on performance decline (6.16 ± 1.58 mg/kg total)	PWPT; hip/back strength; vertical jump; HR; blood lactate; perceived fatigue	CAF 4 mg/kg was ineffective. A single 10 mg/kg dose improved PWPT only before match 1; repeated and selective dosing improved PWPT before matches 3–4, and only selective dosing before match 5. Strength and jump performance were unaffected.
Tzeng et al. (2026)	16 professional male wrestlers; randomized, double-blind crossover	Caffeinated gum (CAF 3 mg/kg) versus placebo gum; chewed for 10 min, followed by a 15 min warm-up before testing	SWPT; handgrip strength; salivary caffeine and alpha-amylase	Caffeinated gum increased throws in both SWPT rounds and in total, and increased post-test salivary alpha-amylase. Handgrip strength was unchanged.
Boxing				
Coswig et al. (2018)	10 male boxing practitioners; double-blind, counterbalanced, crossover	CAF 6 mg/kg versus placebo, 30 min before testing	Boxing match simulations; HR; RPE	CAF raised the interaction time and high effort periods while decreasing pauses in simulated matches. HR and RPE were unchanged.
San Juan et al. (2019)	8 male Olympic-level boxers; randomized, double-blind, placebo-controlled, counterbalanced crossover	CAF 6 mg/kg versus placebo, 75 min before testing	WAnT; CMJ; handgrip strength; blood lactate; lower-limb EMG and neuromuscular efficiency	CAF improved Wingate performance and CMJ height. Neuromuscular efficiency, blood lactate, and handgrip strength were unchanged.
Jodra et al. (2020)	18 male participants (8 Spanish senior national-team boxers and 10 trained-recreational athletes); crossover, randomized, double-blind, placebo-controlled	CAF 6 mg/kg versus placebo, 60 min before testing	WAnT; RPE; mood state; Subjective Vitality Scale	CAF increased Wingate peak and average power and shortened time to peak power in both groups, without increasing RPE. Tension, vigor, and subjective vitality increased only in the elite boxers.
Karate, mixed martial arts, and kickboxing				
Arazi et al. (2016)	10 teenage female karate athletes; double-blind, randomized, and crossover counterbalanced	CAF 2 or 5 mg/kg versus placebo, 60 min before testing	Leg-press 1RM and repetitions at 60% 1RM; vertical jump; RAST; RPE; pain perception	Neither dose improved strength, muscular endurance, vertical jump performance, or RAST power. CAF 5 mg/kg reduced RPE versus both placebo and 2 mg/kg, and reduced pain versus placebo, after the muscular endurance test only.
de Azevedo et al. (2019)	11 male professional mixed martial arts athletes; double-blind, counterbalanced crossover	CAF 5 mg/kg versus placebo, 60 min before testing	Punch frequency and mean and maximal force; readiness to invest physical and mental effort; RPE	CAF did not alter punch frequency, mean or maximal punch force, readiness to invest physical or mental effort, or RPE.
Saremi et al. (2025)	12 professional male kickboxers; double-blind, placebo-controlled, and cross-over	CAF 3 or 6 mg/kg versus placebo, 60 min before testing	Vertical jump; WAnT; maximal treadmill test; time to exhaustion; RPE; muscle soreness immediately and 2 and 12 h after exercise	Both doses increased vertical-jump height and time to exhaustion. CAF 3 mg/kg also increased relative Wingate peak power, whereas 6 mg/kg reduced postexercise muscle soreness; other outcomes were unchanged.

1RM, one-repetition maximum; BJJ, Brazilian jiu-jitsu; CAF, caffeine; CMJ, countermovement jump; EEG, electroencephalography; EFT, Eriksen Flanker Test; EMG, electromyography; ERP, event-related potential; fNIRS, functional near-infrared spectroscopy; FSKT-10s, 10-s Frequency Speed of Kick Test; FSKT-multi, multiple Frequency Speed of Kick Test; HR, heart rate; JGST, Judogi Grip Strength Test; P300, P300 component of the event-related potential; PWPT, Pittsburgh Wrestling Performance Test; RAST, Running-based Anaerobic Sprint Test; RPE, rating of perceived exertion; RPF, rating of perceived fatigue; RPR, rating of perceived recovery; RVIPT, Rapid Visual Information Processing Test; SJFT, Special Judo Fitness Test; s-RPE, session rating of perceived exertion; SWPT, Specific Wrestling Performance Test; TSAT, Taekwondo-Specific Agility Test; UBISP, upper-body intermittent sprint performance; WAnT, Wingate Anaerobic Test.

### 3.1 Studies on combined caffeine protocols

Studies evaluating caffeine as part of combined or mixed-intervention protocols are summarized in **Table 1**. These protocols fell into three broad categories. The first involved caffeine co-administration with sodium bicarbonate, carbohydrate, L-theanine, or Rhodiola rosea. The second paired caffeine with a warm-up or neuromuscular priming strategy, including music, conditioning activity, or post-activation performance enhancement (PAPE). The third comprised alternative mouth-rinse protocols that compared caffeine with carbohydrate or placebo rinses.

Across the included studies, caffeine paired with warm-up music or a conditioning activity yielded the most consistently favorable findings, particularly for taekwondo-specific performance (Delleli et al., 2025, 2024b, a, 2023; Ouergui et al., 2022). Caffeine combined with sodium bicarbonate or PAPE also improved selected outcomes relative to placebo or control conditions, although these combinations did not consistently outperform the corresponding strategy alone (Felippe et al., 2016; Rezaei et al., 2019; Wang et al., 2026; Zhang et al., 2024). Favorable outcomes were also reported for caffeine combined with carbohydrate, L-theanine, or Rhodiola rosea, although each combination was examined in a single study using distinct protocols and outcome measures (Razazan et al., 2025; Suksuwan et al., 2022; Tao et al., 2025). The two mouth-rinse studies produced findings that varied according to the comparator and testing context (Özlükan-Şahin et al., 2024; Pak et al., 2020). Taken together, the current evidence suggests that combined or mixed protocols may provide context-specific benefits, but does not establish a general advantage over isolated caffeine supplementation.

### 3.2 Studies on caffeine supplementation alone

#### 3.2.1 The effects of caffeine on simulated combat performance

In judo, Durkalec-Michalski et al. (2019) reported that 6 and 9 mg/kg increased total SJFT throws and that 9 mg/kg also increased total attacks during three randori bouts. Carmo et al. (2021) further demonstrated that 5 mg/kg caffeine maintained post training SJFT performance after a prolonged judo training session, preventing the decline in throws and fatigue index observed with placebo. By contrast, 5 mg/kg did not alter attack number, effectiveness, efficiency, or neuromuscular performance in another repeated-randori protocol (Saldanha Da Silva Athayde et al., 2019, 2018).

In taekwondo, Santos et al. (2014) observed 5 mg/kg improvements in combat performance, evidenced by increased relative attack times and attack/skipping ratio, with sustained intensity during the second bout. Conversely, Lopes-Silva et al. (2015) reported no improvement with 5 mg/kg in attack number or time, despite greater estimated glycolytic contribution, echoing the results of Sun et al. (2022).

In Jiu-Jitsu, 3 mg/kg increased the frequency and duration of high-intensity offensive actions during simulated Brazilian Jiu-Jitsu competition (Diaz-Lara et al., 2016b). However, the same dosage did not alter the number of offensive or defensive technical actions during traditional jiu-jitsu combat in another study (Merino-Fernández et al., 2022).

In wrestling, Negaresh et al. (2018) investigated caffeine administration strategies during a simulated five-match tournament. 4 mg/kg did not improve PWPT performance, whereas 10 mg/kg enhanced performance only before the first match. In comparison, repeated caffeine dosing (5 × 2 mg/kg) improved performance before the third and fourth matches, while individualized selective dosing based on performance decline maintained performance improvements through the later matches, including the final bout.

In boxing, 6 mg/kg increased the duration of high-intensity activity during simulated rounds (Coswig et al., 2018).

Overall, caffeine supplementation showed potential benefits for simulated combat performance, but effects varied across combat disciplines, match structures, and supplementation protocols.

#### 3.2.2 The effects of caffeine on SJFT performance

The Special Judo Fitness Test (SJFT) is a judo-specific physical performance test in which athletes are required to perform a series of Ippon-Seoi-Nage throws on two partners positioned 3 meters apart. The test comprises three bouts: the first lasting 15 seconds, and the second and third lasting 30 seconds each, with 10-second rest intervals in between. A higher total number of throws across the three bouts indicates better performance. The SJFT index is calculated using the formula: Index = (HR immediately after + HR one minute after)/total number of throws. A lower SJFT index reflects superior judo-specific fitness and faster recovery capacity (Miarka et al., 2011).

Caffeine at 4 mg/kg improved SJFT performance and lowered the SJFT index in young judokas (Astley et al., 2017), with 6 and 9 mg/kg in highly trained male judokas (Durkalec-Michalski et al., 2019), and with 5 mg/kg when post-training performance was compared with placebo (Carmo et al., 2021). However, among habitual caffeine users, significant improvements were observed only with the higher dose of 9 mg/kg.

Conversely, Lopes-Silva et al. (2014) noted that 6 mg/kg failed to improve SJFT performance after a 5-day rapid weight loss. Filip-Stachnik et al. (2021) likewise found no improvement in throws or SJFT index with 2.7 or 5.4 mg/kg delivered as caffeinated chewing gum.

Overall, caffeine-related improvements in SJFT performance were mainly observed with capsule-based supplementation (4–9 mg/kg), whereas effectiveness appeared reduced after rapid weight loss or when caffeine was delivered through chewing gum.

#### 3.2.3 The effects of caffeine on strength, power, and muscular endurance

Findings for anaerobic power were mixed. In elite judokas, caffeine at 5 mg/kg increased Wingate Anaerobic Test (WAnT) peak and mean power during morning testing, whereas the same dose did not affect WAnT performance during afternoon testing (Souissi et al., 2012, 2015). Favorable WAnT outcomes were also reported after 200 mg in taekwondo athletes (Sun et al., 2022) and after 6 mg/kg in boxers (Jodra et al., 2020; San Juan et al., 2019). In kickboxers, 3 mg/kg increased relative WAnT peak power (Saremi et al., 2025).

Beyond WAnT performance, several studies reported improvements in maximal strength, explosive power, and muscular endurance. Caffeine at 3 mg/kg improved handgrip strength, CMJ performance, static-lift duration, and selected bench-press outcomes in Brazilian jiu-jitsu athletes, although these benefits diminished across successive combats (Diaz-Lara et al., 2016a; b). The same dose improved selected bilateral and unilateral CMJ outcomes in traditional jiu-jitsu athletes (Merino-Fernández et al., 2021). Caffeine at 5 mg/kg increased judogi strength endurance and maximal handgrip strength in judo and Jiu-jitsu athletes (Lopes-Silva et al., 2022). In judokas, 3 and 6 mg/kg improved selected bench-press and bench-pull velocities and dynamic judogi-grip repetitions, but did not improve jump height, maximal handgrip strength, or isometric judogi-grip duration (Krawczyk et al., 2022).

By contrast, several studies found no improvement in specific strength or power outcomes. In kickboxers, 6 mg/kg did not improve WAnT power, although both 3 and 6 mg/kg increased vertical-jump height (Saremi et al., 2025). Caffeine at 5 mg/kg did not improve upper-body intermittent sprint power in wrestlers (Aedma et al., 2013). Hip and back strength and vertical-jump performance were also unchanged across the caffeine-dosing protocols examined by Negaresh et al. (2018). In two judo protocols, caffeine at 5 mg/kg did not improve CMJ performance, handgrip, judogi-grip, or upper-limb power (Carmo et al., 2021; Saldanha Da Silva Athayde et al., 2018). Handgrip strength was also unchanged after caffeinated-gum ingestion in wrestlers, despite improved wrestling-specific performance (Tzeng et al., 2026). In adolescent karate athletes, neither 2 nor 5 mg/kg improved leg press strength, muscular endurance, CMJ performance, or running-based anaerobic sprint power (Arazi et al., 2016). Punch force was likewise unchanged after 5 mg/kg in professional mixed martial arts athletes (de Azevedo et al., 2019).

Overall, caffeine produced favorable strength or power outcomes most frequently within the 3–6 mg/kg range, although higher doses may provide benefits under specific conditions.

#### 3.2.4 The effects of caffeine on RPE, HR, and blood lactate

Perceived exertion was generally unchanged following caffeine supplementation, although reductions were reported in selected studies. Lopes-Silva et al. (2014) and Astley et al. (2017) observed lower RPE in judokas, while similar reductions were reported by Ouergui et al. (2023) in elite taekwondo athletes and Arazi et al. (2016) in female karate athletes. In contrast, Diaz-Lara et al. (2016b) reported increased perceived exertion in Brazilian jiu-jitsu athletes.

Heart-rate responses were largely unaffected by caffeine supplementation, although four studies reported increased HR following caffeine ingestion (Aedma et al., 2013; Durkalec-Michalski et al., 2019; Merino-Fernández et al., 2022; Negaresh et al., 2018).

Blood lactate responses were generally characterized by increases following caffeine supplementation (Aedma et al., 2013; Carmo et al., 2021; Diaz-Lara et al., 2016b; Lopes-Silva et al., 2015, 2014; Negaresh et al., 2018; Saldanha Da Silva Athayde et al., 2018; Santos et al., 2014), although several studies reported no significant changes (Filip-Stachnik et al., 2021; Lopes-Silva et al., 2022; Sun et al., 2022).

## 4 Discussion

This review synthesized evidence from 43 reports examining acute caffeine supplementation in combat sport athletes, including both isolated caffeine supplementation and caffeine based combined or mixed intervention protocols. Overall, caffeine showed potential ergogenic effects on selected strength, power, judo specific fitness, and sport specific performance outcomes; however, responses were not consistent across disciplines, tasks, or supplementation strategies. By extending the literature search and integrating both isolated and combined protocols, this review provides a contextual synthesis that considers sport specific demands, participant characteristics, delivery forms, weight cutting status, and tournament structures. Therefore, the present findings complement rather than replace previous meta analyses by providing a more contextual understanding of how caffeine may influence performance under different combat sport conditions.

### 4.1 Factors influencing the variability of caffeine responses

Dose, timing, and delivery form are important factors that may contribute to variability in caffeine responses. Most capsule reports used 3–6 mg/kg approximately 60 min before testing, and both favorable and null results occurred within this range. Higher single doses (9 to 10 mg/kg) benefited selected later bout outcomes in a small number of male wrestling or judo protocols, but these doses may aggravate dehydration index and gastrointestinal symptoms (Negaresh et al., 2018; Durkalec-Michalski et al., 2019). Circadian rhythms have a potential impact on athletic performance. Mora-Rodríguez et al. (2015) observed that caffeine intake in the morning can enhance performance, but found no such improvement in the afternoon, and noted that there were greater side effects in the afternoon. Boyett et al. (2016) also reported that the benefits of caffeine supplementation on individual cycling performance were higher in the morning compared to the evening. Similar patterns have been reported in combat sports, although current evidence is limited to judo athletes (Souissi et al., 2012, 2015). Chewing gum can shorten the pre exercise interval, whereas mouth rinsing may be useful when ingestion is undesirable; however, combat sport specific evidence remains limited. For example, caffeinated gum at doses of 200 or 400 mg did not improve performance in elite judo athletes (Filip-Stachnik et al., 2021), whereas 3 mg/kg caffeine gum improved sport specific performance in wrestlers (Tzeng et al., 2026).

Beyond caffeine related protocols, athlete characteristics may also contribute to variability in caffeine responses. Habitual caffeine consumption may be one possible explanation for the inconsistent findings. For instance, two studies (Saldanha Da Silva Athayde et al., 2019, 2018) that reported no increase in attack frequency among judo athletes involved participants who regularly consumed moderate amounts of caffeine throughout the day, suggesting that habitual intake may reduce responsiveness to acute caffeine supplementation in some athletes. While some research indicates that the ergogenic effects of caffeine can persist over 20 consecutive days of supplementation, these effects may gradually decline compared with the initial response (Lara et al., 2019). Conversely, Sabol et al. (2019) suggested that the acute effects of caffeine may not be significantly influenced by habitual intake. Current combat sport evidence is insufficient to determine whether habitual caffeine intake meaningfully alters acute caffeine responsiveness. Genetic polymorphisms, particularly in *CYP1A2* and *ADORA2A*, have been proposed as potential contributors to inter-individual variability in caffeine responses. However, the evidence synthesized in this review does not allow conclusions about whether these variants contributed to the heterogeneous findings observed in combat sports (Pickering and Kiely, 2018).

Sex related differences may also contribute to variability in caffeine responses, although research on caffeine supplementation in female athletes remains limited compared with their male counterparts (Goldstein et al., 2010). Some evidence suggests that men may demonstrate greater improvements in anaerobic performance following caffeine intake, including strength and speed related outcomes (Mielgo-Ayuso et al., 2019). These differences may be partly related to variations in muscle mass, caffeine metabolism, and physiological responses between sexes (Woolf et al., 2008; Pickering, 2019). While male athletes continue to represent a large proportion of combat sport participants, more research involving female combat sport athletes is needed to clarify whether caffeine responses differ between sexes.

Athlete training level may also contribute to variability in caffeine responses. The conflicting results observed in taekwondo competitions may be associated with differences in athletic proficiency (Pickering and Grgic, 2019). In two studies that did not demonstrate combat performance enhancement, participants were high level elite athletes, including members of the Hong Kong national taekwondo team who trained 11 hours per week and athletes competing at national or international levels who trained 15.5 hours per week (Lopes-Silva et al., 2015; Sun et al., 2022). This training duration was considerably greater than that reported in a study (Santos et al., 2014) showing improved simulated competition performance (4 hours per week). Highly trained athletes may have smaller performance margins for further improvement, potentially reducing the observable ergogenic effects of caffeine. Interestingly, Ouergui et al. (2023) suggested that elite athletes may experience greater benefits from caffeine during taekwondo specific tasks compared with sub elite athletes. This finding is consistent with Collomp et al. (1992), who reported greater performance improvements following caffeine ingestion in well trained athletes compared with amateur athletes. Therefore, training status should be considered when interpreting caffeine related performance changes, particularly in elite athletes with limited room for further improvement.

Pre competition physiological status may also contribute to variability in caffeine responses, particularly in weight category combat sports where rapid weight loss is common. Rapid weight loss has been shown to negatively affect psychological and physiological status, as well as athletic performance. Substantial evidence indicates that weight reduction can impair both aerobic and anaerobic capacity (Franchini et al., 2012). Rapid weight loss may also alter hydration status, substrate availability, sleep quality, and perceived effort, which could contribute to variability in caffeine responses. However, these factors should be considered as potential explanations for heterogeneity rather than established determinants of caffeine effectiveness.

### 4.2 Practical implications

For athletes and practitioners, a practical approach is to evaluate individual responses to caffeine during training before competition. A dose of 3 to 6 mg/kg approximately 60 min before competition or testing represents the most frequently studied strategy, particularly in capsule based protocols. However, dose selection should consider the athlete’s experience, competition demands, and previous response rather than applying a universal recommendation. Current evidence suggests that caffeine may be particularly useful for improving strength, power, repeated high-intensity efforts, and selected fitness-test outcomes. Relatively consistent favorable findings were observed for judo-specific fitness tests and selected taekwondo-specific tasks, although null findings were also reported in both disciplines (**Tables 1**, **2**). Because judo and taekwondo were also among the most frequently studied disciplines, comparisons with less studied combat sports remain tentative. Effects on technical and tactical outcomes remain less consistent and require further investigation.

For same day multiple bouts, practitioners should plan caffeine timing according to the expected competition schedule and consider prior caffeine exposure and total daily intake. Repeated or selective dosing strategies may be useful in specific tournament scenarios but should currently be considered individualized approaches rather than routine practice. The limited evidence from wrestling suggests that adjusting caffeine administration across successive bouts may have the potential to maintain performance, although further research is required before practical recommendations can be established (Negaresh et al., 2018). Caffeinated gum may be useful when the available pre bout interval is short because it allows rapid caffeine absorption through the oral mucosa, whereas caffeine mouth rinsing remains an alternative approach that requires further validation in combat sport settings.

For combined or mixed intervention strategies, future research should focus on determining whether caffeine provides additional benefits beyond the accompanying intervention. Current evidence from caffeine combined with music, conditioning activity, sodium bicarbonate, PAPE, carbohydrate, L theanine, and Rhodiola rosea suggests potential benefits in selected outcomes, but most protocols have been investigated in single studies or small samples. Future trials should therefore include direct comparisons between caffeine alone, the combined strategy, and the individual non caffeine intervention to clarify whether combined strategies provide additional benefits beyond the individual components.

Future studies should quantify habitual caffeine intake and withdrawal status and record adverse events and sleep responses. Comparisons should also consider sex, performance level, training phase, and competitive experience. Further research within the same discipline is needed because boxing, mixed martial arts, BJJ, karate, judo, taekwondo, and wrestling differ in effort duration, intensity distribution, technical demands, scoring systems, and underlying motor requirements.

### 4.3 Limitations

Several limitations should be acknowledged. Although this review incorporated searches from multiple databases, including Web of Science Core Collection, Scopus, PubMed, and SPORTDiscus, some relevant studies may not have been identified. In addition, substantial heterogeneity existed across studies in terms of combat sport disciplines, performance outcomes, caffeine doses, delivery forms, and experimental protocols. This heterogeneity, together with the limited number of studies investigating caffeine based combined or mixed intervention protocols, restricted direct comparisons and prevented quantitative synthesis of the available evidence. Across the evidence base, randomized, double-blind, placebo-controlled crossover designs were common. However, sample sizes were generally small, and the reports varied in comparator structure and in the reporting of potentially relevant caffeine-related moderators. Because methodological quality was not formally graded, the findings should be interpreted as contextual patterns rather than ranked estimates of evidence certainty.

## 5 Conclusions

Acute caffeine supplementation may improve selected strength, power, sport specific fitness, and performance outcomes in combat sport athletes, but benefits are not uniform across disciplines, tasks, or individuals. Doses of 3 to 6 mg/kg represent the most frequently studied protocols, but individual responses should be assessed during training before competition. Evidence for high or repeated doses, rapid delivery forms such as caffeinated gum and mouth rinses, and combined strategies remains preliminary. Future research should further clarify optimal caffeine strategies and individual factors influencing responsiveness in combat sport athletes.
